# Biotics and Children’s and Adolescents’ Health: A Narrative Review

**DOI:** 10.3390/children11030329

**Published:** 2024-03-09

**Authors:** Evangelia Xenopoulou, Ioanna Kontele, Theodoros N. Sergentanis, Maria G. Grammatikopoulou, Milia Tzoutzou, Konstantinos Kotrokois, Artemis Κ. Tsitsika, Tonia Vassilakou

**Affiliations:** 1 Laboratory of Epidemiology, Health Determinants and Well-Being, Department of Public Health Policy, University of West Attica, 115 21 Athens, Greece; evaxeno@gmail.com (E.X.); ikontele@uniwa.gr (I.K.); tsergentanis@uniwa.gr (T.N.S.); kkotrokois@uniwa.gr (K.K.); 2 Department of Rheumatology and Clinical Immunology, Faculty of Medicine, School of Health Sciences, University of Thessaly, Biopolis, 411 10 Larissa, Greece; mgrammat@uth.gr; 3 Laboratory of Nutrition and Dietetics, Department of Nutrition and Dietetics, Harokopio University, Elefteriou Venizelou 70, 176 76 Athens, Greece; miltzou@hua.gr; 4 Adolescent Health Unit, Second Department of Pediatrics, “P. & A. Kyriakou” Children’s Hospital, National and Kapodistrian University of Athens, 115 27 Athens, Greece; info@youth-health.gr

**Keywords:** probiotics, prebiotics, synbiotics, postbiotics, metabiotics, parabiotics, infants, children, adolescents, health conditions, pediatric nutrition

## Abstract

Recently, there has been an increasing interest in the association of gut microbiota with health conditions and the potentially beneficial role of several types of biotics in several population groups, including children and adolescents. Children and adolescents comprise a unique population group due their rapid growth rates, high nutritional requirements, the immaturity of their immune system in early life, and their susceptibility to infectious diseases. The aim of the present study is to investigate the role and limitations of the administration of biotics in specific conditions affecting children and adolescents. A narrative review of related articles published on PubMed up to October 2023 was conducted. The administration of biotics has been evaluated in several health conditions among children and adolescents, such as the treatment and prevention of infectious diarrhea, the prevention of diarrhea after the use of antibiotics, the prevention of necrotizing enterocolitis, the treatment of functional gastrointestinal diseases, such as infant colic, functional abdominal pain, and irritable bowel syndrome, the eradication of *H. pylori*, the treatment of ulcerative colitis and pouchitis, and the prevention of atopic dermatitis, and the findings indicate improved symptoms and various beneficial health outcomes. However, some limitations have been identified regarding probiotics’ use. In conclusion, biotics may have a beneficial impact in several health conditions among children and adolescents. There is a need for additional randomized, controlled clinical studies on the effects of the administration of biotics in children and particularly in adolescents and young adults.

## 1. Introduction

Although ancient Greek physician Hippocrates had reported that “all diseases begin in the gut” 2500 years ago, the correlation between health and the gut microbiota was proposed again in 1907 by a Russian Nobel-awarded scientist, Ellie Metchinkoff, who hypothesized that replacing “septic” gut bacteria with lactic acid-producing bacteria could contribute to normal gut function, as well as a prolonged lifespan [1].

The microflora’s microbes colonize almost every surface of the human body that is exposed to the external environment, such as the skin, the urogenital tract, the mammary gland, the respiratory tissues, and the gastrointestinal tract [2]. A plethora of microorganisms colonize the human intestinal tract and support various physiological functions. The colonization of the gut initiates at birth and continues throughout the first years of life, resulting in a unique gut microbiome for every person. This process promotes the formation of a natural immunological barrier between the host and the environment, thereby assisting the intestinal tract in remaining disease free [3].

The composition of the microbiome varies depending on prenatal events, delivery methods, infant nutrition, infant development environment, and the use of antibiotics [4]. Although previous studies have suggested that the gut microbiota develops an adult-like structure by the age of three years, new research suggests that development may take longer [5].

The use of probiotics, prebiotics, or fermented dietary products has been proposed in order to modify the gut microbiota for the prevention and treatment of many of the above-mentioned disorders [6].

Although they are not components of the host’s microbiome, probiotics are live microorganisms which have a beneficial effect on the host’s health when administered in sufficient quantities [7]. Probiotics include lactic acid-producing bacteria, i.e., *Lactobacillus* and *Bifidobacterium* species, as well as *Saccharomyces boulardii* yeast, which are found both in foods and dietary supplements [8]. Health-promoting properties, such as the prevention and treatment of antibiotics-associated diarrhea and diarrhea caused by rotaviruses and the prevention of allergies, have been attributed to certain probiotic products [9].

The definition of prebiotics is more recent, and they were initially described by Gibson and Roberfroid in 1995 as “non-digestible food ingredients that beneficially affect the host by selectively stimulating the growth and/or activity of one or a limited number of bacteria already resident in the colon” [10]. In 2017, the International Scientific Association for Probiotics and Prebiotics (ISAPP) published a consensus statement that updated the definition of a prebiotic to “a substrate that is selectively utilized by host microorganisms conferring a health benefit” [11]. The most common prebiotics are oligofructose, inulin, lactulose, galacto-oligosaccharides, and oligosaccharides of breast milk [11]. Prebiotics may be used either as an alternative to probiotics or to enhance their action.

Synbiotics were recently defined as “a mixture comprising live microorganisms and substrates selectively utilized by host microorganisms that confers a health benefit on the host”. Synbiotics may be formulated into foods, drugs, or supplements. The main beneficial properties of synbiotics for the host include the improvement in the implantation and survival of live microbial dietary supplements in the gut, as they may selectively stimulate the growth and/or activate the metabolism of one or more health-promoting bacteria [12].

Due to safety concerns raised regarding probiotics’ use, new biotics are being developed, such as postbiotics and parabiotics.

Metabiotics, or postbiotics, are metabolites (substances produced by microorganisms such as probiotics) with direct and indirect benefits to the host [13]. They are not viable organisms, but they carry the beneficial substances produced by microorganisms. Postbiotics include a variety of metabolic by-products of live probiotic bacteria. Some comparative studies between pro- and postbiotics conducted in vitro and in vivo suggest that postbiotics may have similar potential health benefits to probiotics [14,15,16].

Parabiotics, also known as paraprobiotics, inactivated probiotics, or “ghost probiotics”, contain microorganisms deliberately inactivated to lose their viability and are “non-viable intact or ruptured microbial cells or crude cell extracts, as intact or ruptured containing cell components of probiotic cells upon lysis, which when administered orally or topically in adequate amounts, are considered to confer a health benefit on the human or animal consumer” [13].

The aim of the present review is to investigate the health benefits and limitations related to biotics’ administration in specific health conditions commonly affecting children and adolescents as well as the new trends regarding their use.

## 2. Materials and Methods

An electronic search of the international literature was conducted in the PubMed database, including results published until October 2023, in order to identify relevant observational studies, randomized controlled trials, reviews, and systematic reviews with or without meta-analyses.

The search strategy was based on the combination of the following keywords (probiotics OR prebiotics OR synbiotics OR postbiotics OR metabiotics OR parabiotics) AND (infants OR children OR adolescents) AND “health conditions”. The reference list of all relevant articles was also thoroughly examined, and possibly relevant articles were hand-searched. Significant attention was paid to the most recent articles, as well as to systematic reviews and meta-analyses. The articles were assessed according to their title, abstract, and full text, and the most relevant articles were reviewed. Relevant organization reports and guidelines were also been examined. Studies involving adult patients were excluded, as well as articles published before 2010 and/or in other languages than English and Greek.

## 3. Results

Selected studies on the administration of biotics regarding several health conditions of children and adolescents are presented in Table 1:

### 3.1. Effectiveness of Probiotics, Prebiotics and Synbiotics in Children’s and Adolescents’ Health

The effectiveness of the administration of various probiotic strains in children and adolescents has been evaluated in the following conditions: the treatment and prevention of infectious diarrhea, the prevention of diarrhea after the use of antibiotics, the prevention of necrotizing enterocolitis, the treatment of functional gastrointestinal diseases, such as infant colic, functional abdominal pain, and irritable bowel syndrome, the eradication of *H. pylori*, the treatment of ulcerative colitis and pouchitis, and the prevention of atopic dermatitis.

### 3.2. Probiotics and Acute Diarrhea in Children

The ESPGHAN (European Society for Pediatric Gastroenterology, Hepatology and Nutrition) recommends the use of *Lactobacillus* GG and *S. Boulardii* adjunctively in the treatment of children with acute diarrhea. The guideline considers only the specific species and in specific dosages [51]. The recommendations are based on reviews of meta-analyses, such as a review published in 2010 in the Cochrane Database where the main finding was a reduction of 25 h in the mean duration of diarrhea and 29 h in diarrhea due to rotavirus infection [17]. However, *Lactobacillus reuteri* species, although well tolerated in children with acute gastroenteritis, have very low efficacy in the management of acute diarrhea [18]. Also, the combined administration of *L. helveticus* and *L. rhamnosus* species does not provide benefits in the management of acute gastroenteritis [19]. The Global Recommendations of the World Gastroenterology Organization (2017) are also harmonized with the above guidelines regarding the types of probiotics administered to treat acute diarrhea in children [52]. On the other hand, the AGA (American Gastroenterological Association) does not recommend the administration of probiotics in acute gastroenteritis, arguing that the ESPGHAN recommendations preceded the publication of two large multicenter studies from North America, which were available later [53].

### 3.3. Probiotics and Nosocomial Diarrhea in Children

Hospital-acquired infections have numerous adverse effects on patients, their families, and the health system. In developed countries, the incidence of nosocomial diarrhea is high, despite the implementation of preventive measures. Gastrointestinal infections account for the majority of nosocomial infections among children, and rotavirus is still a very serious pathogen [54]. The introduction of probiotics in order to prevent nosocomial diarrhea is reported as an effective measure, in combination with vaccination, hand hygiene, patient isolation, and inpatient restraint [55,56]. A significant number of randomized controlled trials have studied the possible preventive effect of probiotics’ use on nosocomial diarrhea [20,21]. Current evidence and a systematic literature review have shown that the introduction of *Lactobacillus rhamnosus* GG may reduce the risk of nosocomial diarrhea. These are the recommendations of the Working Group on Probiotics and Prebiotics of ESPGHAN [57]. The Global Recommendations of the World Gastroenterology Organization (2017) also harmonize with the above results regarding the types of probiotics used to treat nosocomial diarrhea in children [52].

### 3.4. Probiotics, Prebiotics, and Antibiotics-Associated Diarrhea Prevention in Children

Antibiotics-associated diarrhea is a common adverse effect of antibiotics’ administration, occurring equally in hospitalized and non-hospitalized patients and affecting one-third of patients receiving antibiotics [58]. The consumption of probiotics and/or fermented food products has been reported as a preventive measure against antibiotic-induced diarrhea [59]. Several reviews and meta-analyses have reported the preventive effect of probiotics’ use for the prevention of diarrhea associated with antibiotics administration [22,23]. The recommendations of the ESPGHAN regarding the use of probiotics for antibiotics-associated diarrhea prevention in children are also based on systematic reviews and randomized controlled trials, and they recommend the use of *Lactobacillus rhamnosus* GG or *Saccharomyces boulardii* for the prevention of diarrhea due to *Clostiridium difficile* infection. Although other species and their combinations have been examined, there is no strong evidence for their effectiveness [51]. The recommendations of the American Gastroenterological Association (AGA) are consistent with those of ESPGHAN, but further study in this area is recommended [53]. The Global Recommendations of the World Gastroenterology Organization (2017) align with the above regarding the types of probiotics to be used. Finally, an ESPGHAN report on the use of inulin and fructo-oligosaccharides in 2012 showed that they were not effective treatments [60].

### 3.5. Probiotics and Eradication of Helicobacter pylori in Children

The infection caused by *Helicobacter pylori* is a common condition, occurring in 50% of the world’s population. Its colonization may result in the appearance of various gastrointestinal conditions [28]. Several studies have reported that traditional *H. pylori* eradication treatments have less than 80% effectiveness, while their use comes with the side effects associated with antibiotics’ use [61]. Some recent studies report that the application of specific probiotic treatments may improve the efficacy and resistance of *H. pylori* treatment [24]. A meta-analysis of randomized controlled trials concluded that, during *H. pylori* eradication therapy, the administration of probiotics may improve the degree of eradication, reducing the incidence of treatment adverse effects and alleviating clinical symptoms associated with the disease. However, the interpretation of the results of this meta-analysis should be undertaken with caution due to the presence of heterogeneity among the included trials [25]. Another meta-analysis concluded that the supplemental use of specific types of probiotics (i.e., *Lactobacillus acidophilus*/*Bifidobacterium animalis* and an eight-strain mixture) may improve the eradication rate of *H. pylori* and prevent side effects and diarrhea associated with antibiotics; however, not all strains of probiotics are effective [26]. The World Gastroenterology Organization (2017) recommends the use of *Saccharomyces boulardii* and *Lactobacillus casei* in order to reduce side effects and improve the effectiveness of therapy [52].

### 3.6. Synbiotics and Eradication of Helicobacter pylori in Children

A randomized prospective double-blind controlled trial on the use of synbiotics concluded that eradication was significantly higher in the synbiotic group than in the placebo group. Treatment adverse effects, e.g., diarrhea, nausea, vomit, epigastric pain, constipation, taste disturbances, and flatulence were the same in both groups. However, anorexia was significantly improved in the group receiving synbiotics [27]. A systematic review and meta-analysis published in 2019 reported that probiotics may improve the degree of *H. pylori* eradication and reduce treatment side effects. However, these findings need further confirmation from future larger studies [28].

### 3.7. Probiotics and Infant Colic

Infant colic is defined as “intense crying for at least three hours a day, at least three days a week, for at least three weeks” [62]. Infantile colic affects a significant number of infants worldwide and causes concerns among their families. The symptomatology is wide and general, and, although not indicative of a disease presence, in a small number of infants, it may highlight a serious underlying condition that may require medical evaluation [63]. The most commonly used probiotics in infant colic are *Lactobacillus*, *Bifidobacterium*, and *Streptococcus*. There is a growing amount of evidence that the microbial flora of colicky infants differs from that of non-colicky infants, and it appears that probiotics may improve intestinal balance and provide a healthier microbiome, suggesting a potential preventive role of probiotics for infant colic [29].

A review of randomized controlled trials published in the Cochrane Database of Systematic Reviews in 2019 reported that there is no clear evidence that the use of probiotics, prebiotics, and synbiotics prevents colic in infants compared to infants who received a placebo, although it was associated with reduced daily crying time. No clear differences in adverse effects emerged. The researchers suggested the need for further research [29]. The recommendations of the European Pediatric Association (2018) indicate the use of *L. reuteri* DSM 17938 strain for the management of colic in breastfed infants, while the effectiveness for colic prevention is low [64]. The international recommendations of the World Gastroenterology Organization (2017) harmonize with the above recommendations for infant colic [52].

### 3.8. Probiotics and Functional Gastrointestinal Disorders in Children

Regarding functional gastrointestinal disorders in children, probiotics have been studied for the treatment of irritable bowel syndrome (IBS) as well as for functional abdominal pain not otherwise specified. Irritable bowel syndrome should be considered as a condition in cases of abdominal pain present for at least four days per month which is combined with a significant change in bowel movement and/or stools appearance [65]. Functional abdominal pain is defined as “pain that occurs at least four times a month and includes episodes of pain or ongoing abdominal pain isolated under normal conditions and which, after appropriate evaluation, cannot be attributed to another clinical condition” [66]. Both disorders are common and affect approximately one-third of school-aged children. Moreover, due to the largely unexplained etiology, no usual treatment is available [67]. As microbiome alteration is one of the findings in this condition, probiotics have been proposed as one of the standard therapeutic methods [30].

According to a review by the American Gastroenterology Association (AGA) (2018), there is evidence that the administration of probiotics might reduce the intensity of functional abdominal pain in children. Only two species (LGG and *L. reuteri* DSM 17938) have been shown to exhibit a positive effect in more than two randomized controlled trials. The AGA was unable to issue recommendations due to limitations regarding current evidence and the absence of available guidelines [64]. The international recommendations of the World Gastroenterology Organization (2017) include the use of specific combinations of probiotic bacterial strains at specific doses in irritable bowel syndrome in children [52]. In 2020, the AGA recommended the use of probiotics in children with symptoms of irritable bowel syndrome only in the context of a clinical trial [53].

### 3.9. Prebiotics and Irritable Bowel Syndrome in Children and Adolescents

A review of systematic reviews and meta-analyses published in 2019 concludes that the benefits of using prebiotics to treat irritable bowel syndrome in children remains theoretical, despite promising results during preclinical trials [31]. It is worth noting, however, the high rate of improvement in functional abdominal pain associated with gastrointestinal disorders in response to placebo administration. This rate reaches 41%, as shown by systematic reviews and randomized controlled clinical trials conducted in a group of children and adolescents aged 4–18 years old [68].

### 3.10. Probiotics and Ulcerative Colitis in Children

The American Gastroenterology Association recommends the use of probiotics only within a clinical trial in children. Despite the growing interest, available data are limited mainly due to studies’ design, patient populations, and the heterogeneity in the specific probiotics studied [53]. The international recommendations of the World Gastroenterology Organization include the use of specific combinations of probiotic bacterial strains in specific doses and in addition to the established therapeutic models [52].

### 3.11. Probiotics and Crohn’s Diseases in Children

The recommendations issued by the American Gastroenterology Association include the administration of probiotics in children with Crohn’s disease only in the context of clinical trials. The changes observed in the gut of Crohn’s disease patients are being increasingly investigated, and there is a growing interest in microbiome-based therapies, such as probiotics use and fecal microbiome transplantation. Studies’ results on the administration of probiotics in Crohn’s disease are limited mainly due to small sample sizes, as well as the heterogeneity of patient populations and differences in probiotic combinations tested [53].

### 3.12. Probiotics and Pouchitis in Children

The American Gastroenterology Association only recommends the administration of a combination of eight probiotic strains (namely *L. paracasei* subsp. *Paracasei*, *L. plantarum*, *L. acidophilus*, *L. delbbrueckii* subsp. *Bulgaricus*, *B. longum* subsp. *Infantis*, and *S. salivarius* subsp. *thermophiles*) and no other or more probiotics in children with pouchitis. Pouchitis is a common postoperative complication after operations for ulcerative colitis. The gut microbiome has been implicated as providing some contribution to the condition’s pathogenesis. The potential use of microbiome therapy for pouchitis has been recommended [53].

### 3.13. Probiotics and Necroticizing Enterocolitis in Infants

Necrotizing enterocolitis is a common, critical gastrointestinal disease among premature infants. The severity of the disease shows great variation. Recent data have reported a strong association between intestinal microbiota dysbiosis and the occurrence of necrotizing enterocolitis [69]. The American Gastroenterology Association recommends the use of a specific probiotic strain or combination of strains to prevent necrotizing enterocolitis in preterm infants with a gestational age of less than 37 weeks, as well as in low-birth-weight neonates [53].

According to the World Gastroenterology Organization (2017), meta-analyses of randomized controlled trials have also indicated a reduction in the death risk in infant groups given probiotics. However, not all probiotics examined were found to be effective, and it is not recommended to routinely use probiotics in preterm units due to limited data [52].

### 3.14. Probiotics, Prebiotics, Synbiotics, and Allergy Prevention in Children

The significant increase in the incidence of allergic diseases and asthma is inevitably associated with complex changes in both the environment and the lifestyle. Urbanization, as well as the global reduction in environmental biodiversity, are directly linked with changes in the human microbiome that are crucial for the maturation of the immune system and its functions [70]. Ecological pressures on microbiome diversity indicate the differentiation of individual exposure to factors such as dietary habits (i.e., increased consumption of processed foods, decreased intake of fresh and fermented foods), physical inactivity (sedentary lifestyle), indoor living (insufficient vitamin D formation, less contact with the natural environment and limited exposure to environmental biodiversity), and various socio-economic factors that favor “dysbiosis” [71]. “Dysbiosis” is blamed for non-communicable diseases including allergy and the development of eczema and asthma [32].

Environmental conditions during early antigen exposure, a period of gastrointestinal defense system immaturity, are crucial for antigen resistance. Mechanisms that trigger and maintain resistance to food antigens have yet to be determined, but early exposure to microbes appears to be essential for promoting an appropriate, normal environment during this period of dynamic growth [72].

Delivery type, antibiotics’ use, breastfeeding, environmental factors during the perinatal period, and countless factors that affect the mother’s microbiome during the periods of pregnancy and breastfeeding are important factors regarding the early colonization process [33]. For example, there are indications that the reduced microbial diversity of the gut microbiome and increased levels of *Enterobacteriaceae* and *Bacteroidetes* in early life are linked with an increased risk of developing food sensitivities and eczema. In addition, the composition and diversity of the gut microbiome at the age of 3–6 months has been correlated with the resolution of milk allergy at 8 years of age [33].

The introduction of probiotics and prebiotics during pregnancy may affect the gut microbiome of the mother and progressively result in the transfer of resistance factors (e.g., cytokines, antibodies, and growth factors) across the placenta, guiding the development of the fetal defense system [73]. During natural labor, the fetal gut acquires the vaginal microbiome and gut of the mother. The postpartum oral administration of probiotics and prebiotics along with breastfeeding and a high-fiber diet may enhance the continued colonization of the intestine with synbiotic bacteria (including *Clostiridium* spp. and *Bacteroides fragilis*), maintaining homeostasis. This, consequently, may prevent gut inflammation and reduce the risk of food allergies by facilitating IgA immunoglobulin secretion and regulatory T-cell responses. IgA is important for initiating the composition of the gastrointestinal microbiome and may strengthen the intestinal barrier. Accordingly, aberrant IgA responses in the gut microbiome of infants have been observed to precede the development of allergy in childhood up to the age of seven years [74].

A preventive effect of probiotics against eczema but not against other allergic conditions has been supported by meta-analyses. In 2017, the World Allergy Organization recommended the administration of probiotics for pregnant and lactating mothers and infants in cases of high risk for allergy based on hereditary predisposition; however, the question of how long the administration should last is not yet answered [34]. Compared to studies on probiotics, studies on prebiotics regarding allergy prevention are fewer in number. Meta-analyses indicate some benefits of prebiotics’ administration in early respiratory and food allergies. Furthermore, the World Allergy Organization recommends prebiotics’ use for non-exclusively breastfed infants [35]. In particular, the available results should be interpreted carefully due to the bifidogenic effect of lactose and its different content in various formulas (hydrolyzed/amino acid-based formulas), which might be a confounding factor in prebiotic studies. More studies are necessary to address specific guidelines regarding the administration of probiotics, prebiotics, and synbiotics for the prevention of allergic conditions and asthma [33]. The European Academy of Allergy and Clinical Immunology and the ESPGHAN do not recommend the administration of probiotics for the prevention of allergies in children, due to a lack of evidence [33].

### 3.15. Probiotics, Prebiotics, and Synbiotics: Studies of Their Effects on Various Other Diseases and Conditions Affecting Children and Adolescents

Some studies show limited benefits from the administration of probiotics or prebiotics in disorders such as autism spectrum disorders and attention deficit hyperactivity disorder in children, without defined dosage and with multiple different strains and variable durations of administration [36]. Nevertheless, only a few related studies have examined this field, and it has not been clear whether gut dysbiosis is a causal factor or stems from processes that accompany mental disorders. A better understanding of the contribution of specific bacteria to the pathways connecting the gut to the brain in children and adolescents could potentially provide alternative tools for a clinical psychiatrist [37,38].

Recent studies have shown some benefits of probiotics and/or prebiotics in adult obesity; however, there is limited experience concerning children and adolescents. A randomized, controlled study showed positive results after the administration of synbiotics in relation to the levels of lipids and oxidative stress in children with early obesity, with additional benefits in weight loss compared to previous studies [39]. A meta-analysis in relation to weight change in children and adolescents after the administration of probiotics (mainly Lactobacillus), showed that there may be some relationship between probiotics consumption and weight gain in children; however, further studies are needed [40].

A systematic literature review concluded that there is no specific pattern of variation in the fecal microbiome in constipation. Also, despite the positive results in specific characteristics of the intestinal environment, there is still no evidence to recommend probiotics in the treatment of constipation in children and adolescents [41].

Based on a systematic review of randomized controlled trials in ages up to 18 years, it was concluded that probiotics may prevent acute otitis media in children who are not prone to it. The strains, duration, frequency, and initiation time of probiotic administration still need further research [42].

Regarding viral respiratory infections, their wide range of etiological factors pose a challenge for the development of effective treatments. Research shows that probiotics may decrease the risk or duration of symptoms of respiratory infections in children. A systematic review of 33 clinical trials published in 2014 showed that probiotics appear to be useful in the treatment of respiratory diseases either by acting directly against viruses or by stimulating the immune system. However, their action on specific viruses has not been sufficiently investigated. Nevertheless, more research is needed in different population groups on the dosage of administered probiotics, the comparison between different strains/species, and the clarification of the mechanisms of action against viruses [75].

The research surrounding the potential benefits of probiotics, prebiotics, and synbiotics is continuous and concerns various conditions that are potentially linked to the microbiome and dysbiosis. However, many studies fail to prove the benefits of their use, or they do not mention the age of the research participants. For example, a study regarding the administration of orthodontic treatments to reduce malocclusion with positive results does not specify the age of the research population [43].

### 3.16. Safety Issues

Most probiotics used today come from either fermented foods or microbes that colonize a healthy person and have been used in products for decades. Based on the prevention exerted by lactobacilli present in fermented foods as normal colonizers of the human body, and the low level of contamination attributed to them, their potential pathogenicity is considered by experts in the field to be quite low [52].

The ESPGHAN Working Group has not addressed the safety evaluation of probiotics, as this was thoroughly performed in 2011 during a review by the US Agency for Healthcare Research and Quality [76]. Although the use of probiotics is safe in healthy populations, caution should be addressed to specific patient groups. Increased risk for adverse effects has been noted in cases of immunosuppressed individuals, premature infants, life-threatening illnesses, structural heart disease, patients with central catheters, and prospective translocation of probiotics across the gut wall. The risk of unwanted actions is greater in individuals with severe underlying diseases [76].

### 3.17. Metabiotics or Postbiotics: A Step after Probiotics and Prebiotics

Metabiotics include a variety of molecules, such as cell-free supernatant, secreted proteins/peptides, amino acids, neurotransmitters, organic acids, vitamins, short-chain fatty acids, bacteriocins, secreted biosurfactants, flavonoids, terpenoids, phenolic-derived postbiotics, etc. [77,78,79]. They have immunoregulatory, anti-inflammatory, anti-oxidant, and anti-cancer properties [80,81].

Postbiotics allow the intestinal microbiota to act locally as well as remotely, using communication axes between the intestine and target organs [82]. These actions include important effects for the pediatric age like anti-inflammatory, anti-microbial, anti-cancer, and immunomodulating effects, when intestinal immaturity increases vulnerability [45].

According to a recent meta-analysis [46], supplementation with postbiotics can prevent common infectious diseases among children. More specifically, heat-killed *Lactobacillus acidophilus LB* reduces the duration of diarrhea, whereas preventive supplementation with heat-inactivated *L. paracasei CBA L74* reduces the risk of diarrhea, pharyngitis, and laryngitis compared to placebo [46]. Most importantly, postbiotics carry a variety of safety advantages over probiotics, including bifidogenic effects, making them suitable for supplements, even among the most vulnerable, including premature newborns [45,47]. Some of these properties are used in clinical practice [83], and infant formulas containing postbiotics are effective in reducing the severity of acute gastroenteritis, the number of hospitalizations, the incidence of acute dehydration, and the need for medical consultations, as well as the prescriptions required for oral rehydration solutions [48,49].

A recent review by Pique et al. [44] indicates that postbiotics exert several pharmacodynamic features over live bacteria. As they do not contain live microorganisms, the risks associated with their intake are limited.

### 3.18. Parabiotics

The goal of the administration of both probiotics and parabiotics is a positive impact on consumer’s health. Parabiotics maintain their potential beneficial effects on the host at the intestinal level in vivo. It has been reported that postprobiotics have an excellent antagonistic advantage over live probiotic bacteria against enteropathogens [84].

As such, their use as safe alternatives to probiotics has been suggested [85] for critically ill, preterm neonates with compromised gut integrity, as these infants are at greater risk of probiotic sepsis as a result of translocation. Furthermore, according to a strain-specific meta-analysis of *B. breve M-16V* in preterm infants [50], significant benefits were noted regarding mortality, length of hospital stay, and postnatal age at full feeds.

Both postbiotics and parabiotics have various beneficial properties in the host physiology, including anti-inflammatory, anti-oxidant, and immunomodulatory properties; they also inhibit adhesion, biofilm formation, viral infection, and proliferation, exerting also anti-hypertensive and cholesterol-lowering effects.

## 4. Discussion

The present narrative review aimed to investigate and summarize the health benefits and limitations related to the administration of biotics in several health conditions commonly affecting children and adolescents, as well as the new trends regarding their use.

Knowledge surrounding the role of the gut microbiome in health and disease is growing rapidly, and the amount of published scientific research on the benefits of its modulation via the administration of biotics is growing exponentially. The vast majority of microorganisms enhance the symbiotic host–bacteria interactions that are crucial for human health. The gut microbiota is involved in human metabolism, the regulation of energy balance, fat deposition, liver and skeletal muscle function, and the production of metabolites and neurotransmitters. Moreover, it determines the balance between beneficial and harmful chemical transformations in the intestinal lumen and participates in the development, training, and homeostasis of the immune system [86]. Over the past decade, a large amount of research has demonstrated the essential role of the gut microbiota in the developmental programming of newborns, such as their metabolism, immune system development, and cognitive development [87,88,89]. Promoting the development of the microbiota in the first 1000 days of life is critical for lifelong health [90].

It is well known that the early colonization of the gut may influence future disease occurrence [91]. The stability of the microbiome is also very important. Dysbiosis (i.e., the modification of the composition of the gut microbiome) may be associated with various clinical conditions, such as acute and chronic inflammatory bowel diseases, irritable bowel syndrome, allergies, autoimmune diseases, mental disorders, metabolic disorders, and obesity [86,92].

The ability to manage the composition and metabolic footprint of the gut microbiome has been well known for decades through the use of probiotics, prebiotics, and the combination of those two, synbiotics [11]. There are different routes of administration for probiotics, prebiotics, and synbiotics, e.g., dermal, rectal, vaginal, and oral, with the latter being the most common. Regarding oral administration, probiotics need to survive the low pH of the gut and gastric fluids. Therefore, probiotics’ survival in the gastrointestinal tract is usually demonstrated by the microbial strains present in stool samples [93].

Regarding current trends on the different uses of biotics in diseases, the present review highlighted a variety of important fields, such as the administration of probiotics in the context of acute infectious diarrhea [17,51,52], nosocomial diarrhea [20,21,52,57], diarrhea associated with antibiotics [22,23,51,53,59,60], functional abdominal pain [53,64], pouchitis [53], and necrotizing enterocolitis in infants [53], as well as their role as adjuncts in ulcerative colitis [52], in the improvement in the eradication rate of *H. pylori* [24,25,26], and as prevention tools for allergic disease in infants [34,37]. Emerging fields include neurodevelopmental disorders [36,37,38], obesity [40], otitis media [42], and viral respiratory infections [75].

The impact of probiotics on the intestinal ecosystem includes their influence on mucosal defense mechanisms, their interaction with common or potentially pathogenic microbes, the production of metabolic end products such as short-chain fatty acids, as well as the communication with host cells through chemical signaling. These mechanisms may result in antimicrobial action, immune differentiation, the stimulation of mucus production, a reduction in IgA immunoglobulin secretion, and the destruction of toxin receptors [94,95]. The effectiveness of probiotics is strain specific. Their benefits are scientifically proven for their use in selected clinical conditions, and they are not recommended for specific groups of patients. Thus, specific strains are effective in the prevention of antibiotics-associated and nosocomial diarrhea and in the treatment of acute gastroenteritis, the treatment of infantile colic in breast-fed infants, and the prevention of upper respiratory tract infections [64]. However, special care is required in premature infants, those who are immunocompromised, patients with life-threatening illnesses, and those with central venous catheters, valvular heart disease, and short bowel syndrome [64].

Prebiotics are not digestible by the host and lead to health benefits for the individual through a positive influence on beneficial microbes. The intake of prebiotics has been linked to an increased number of beneficial anaerobic bacteria in the large intestine, a decreased population of potentially pathogenic microorganisms, increased calcium absorption, increased stool volume, reduced gastrointestinal transit time, and possibly decreased blood lipids [52].

The development of biotherapeutic patterns that include both microbial strains and prebiotics in combination may lead to improved effects in the small and large intestine. Such combinations of probiotic products may be more effective, and their protective and stimulatory effects may be superior to the administration of their components individually [94].

There are many different debates surrounding the use of probiotics for various clinical conditions (some are not considered pathological, such as colic), as well as the ideal time of use and duration of their administration. Studies regarding specific population groups are scarce [64].

Parabiotics and postbiotics have several advantages over traditional probiotics including their purified nature, the known molecular structure, use in purified forms, the specific mechanisms of action, triggering only a specific downstream pathway, and their ease in large-scale industrial production and storage [96,97]. Despite the supported beneficial effects of probiotics, some limitations of their use have been postulated, along with the need for the further investigation of molecular mechanisms. Different strain-specific behaviors, allochthonous or autochthonous niche-specific actions of probiotics, the potential for antibiotic resistance, virulence genes transfer, and the questionable maintenance of their viability and stability during production have been discussed; moreover, the possibility of bacterial translocation to tissue or blood, opportunistic infections, bacteremia, infective endocarditis, and sepsis in immunocompromised individuals have been raised as concerns [98,99,100]. In addition, the low concentrations of probiotic-generated, biologically active compounds measured in target sites during the traditional application of live probiotics were considered ineffective in vivo [80,101]. On the other hand, several host-specific factors in the gastrointestinal tract (GIT) might affect live probiotics and activate several bacterial genes for the degradation and production of various nutrients [102,103].

Often, gaps are noticed, especially among adolescents, in all areas of health; thus, the issues and problems they face sometimes fall under the purview of pediatric specialties and sometimes under the purview of adult specialties. This results in the inappropriate and non-systematic support of this particularly sensitive population group, where significant and decisive changes occur that can affect the course of the individual’s life. Moreover, probiotics and fermented foods are not synonyms; probiotics are products containing known species of beneficial organisms, whereas fermented foods are prepared in lactic acid fermentation, and there is wide variety of specific strains of bacteria present in fermented foods. Furthermore, probiotics have been designed for specific health needs, whereas fermented food products are often associated with known cultures without the inherent necessity of clinical benefit [104].

Significant gaps in knowledge have been noticed regarding this promising and important area of research due to the marked heterogeneity among studies and the variety of strains studied. The lack of reporting of serious adverse effects makes it difficult to estimate true adverse effects. The lack of detailed knowledge about the production procedure of those products makes the comparisons between them difficult and reduces the ability of patients to choose specific products. This indicates the need to take them only on medical recommendation. Future high-quality studies are urgently needed to fill these gaps, as well as advanced monitoring mechanisms by relevant agencies.

## 5. Limitations

Although a plethora of studies have been conducted regarding the health benefits of probiotics in general, and specifically regarding children and adolescents, the level of evidence of the existing studies does not support the introduction of recommendations in most cases. There are certain limitations regarding the development of recommendations on the use of probiotics, mainly due to the research design, sample size, types and specific strains of probiotics used, and safety issues, or due to the purpose of probiotic administration (prevention or therapy). There is no consensus between scientific societies in of the recommendations for certain health conditions among children and adolescents.

There is a clear need for more randomized, controlled clinical studies on the effects of the administration of probiotics, prebiotics, and synbiotics in children, and particularly in adolescents and young adults (15–20 years), where available data are scarce [105]. Moreover, the safety of the use of biotics in immunocompromised subjects should also be established. Future studies should also examine the stability of new biotics, parabiotics, and postbiotics, along with further mechanistic research.

Limitations in the methodology of the current study should be considered, as this is not a systematic review. Due to this fact, it was not possible to present a PRISMA flow chart with the successive steps of document selection. Moreover, the search was limited only to one literature database, namely PubMed. These issues may have affected the articles included in the review. On the other hand, a thorough effort was undertaken to include the findings of all identified relevant articles and reports, as well as their references on the main health benefits of the use of biotics among children and adolescents.

## 6. Conclusions

There is an increasing interest in the association of the gut microbiota with health conditions and the potentially beneficial role of several types of biotics in several population groups, including children and adolescents. A plethora of studies have highlighted the beneficial effects of probiotics, prebiotics, and synbiotics in various health conditions in the vulnerable population group of children and adolescents. Parabiotics and postbiotics are the evolving products in the functional biotics arena which aim to overcome safety and effectiveness issues associated with the use of probiotics. These have several advantages over traditional probiotics.

However, several gaps have been identified in this promising and important research area. There is a need for additional randomized, controlled clinical studies on the effects of the administration of biotics in children and particularly in adolescents and young adults.

The study of the health benefits and limitations of the use of biotics, in the context of the present literature review, results in the question of whether it is possible to ensure a balance between the benefits for the individual’s health and the profit of the production industry of biotics in such a wide market of products, where the control mechanisms are not sufficiently developed. At the same time, a pertinent question arising under these conditions is whether the intervention in the microbiome can and will be maintained within the fundamental principles of bioethics.

## Figures and Tables

**Table 1:** Selected studies that have evaluated the effectiveness of the administration of various probiotic strains, prebiotics, synbiotics, and metabiotics in specific health conditions in children and adolescents.

Study	Population	Methodology	Diseases/Conditions	Biotics Used	Main Results
Allen et al. (2010) [17]	Adults and children (*n* = 8014), infants and children (*n* = 6489)	Systematic review of randomized and quasi-randomized controlled trials	Acute infectious diarrhea	Various probiotics	Probiotics reduced the duration of diarrhoea. However, the effect size varied significantly between studies.
Szymański et al. (2019) [18]	Children < 5 years (*n* = 100)	Randomized controlled trial	Acute gastroenteritis	*Lactobacillus reuteri*	*L. reuteri* used as an adjunct to rehydration therapy did not reduce the duration of diarrhea compared with placebo; nevertheless, it reduced the duration of hospitalization.
Freedman et al. (2020) [19]	Children (*n* = 816)	Randomized controlled trial	Acute gastroenteritis	*Lactobacillus helveticus*, *Lactobacillus rhamnosus*	No virus-specific beneficial effects were attributable to the probiotic.
Wanke et Szajewska (2014) [20]	Infants, children, and adolescents aged 1 month to 18 years (*n* = 1343)	Systematic review and meta-analysis of randomized controlled trials	Healthcare-associated diarrhea	*Lactobacillus rhamnosus* GG, *Bifidobacterium bifidum*, *Streptococcus thermophilus*, *Lactobacillus reuteri* DSM 17938, *Lactobacillus delbrueckii* H2B20	The administration of *Lactobacillus rhamnosus* GG, as well as *Bifidobacterium bifidum* and *Streptococcus thermophilus*, resulted in a reduction in the healthcare-associated diarrhea risk; however, no effect was found with *Lactobacillus reuteri* DSM 17938 and *Lactobacillus delbrueckii* H2B20.
Szajewska et al. (2011) [21]	Infants, children, and adolescents aged 1 month to 18 years (*n* = 1092)	Meta-analysis	Healthcare-associated diarrhea	*Lactobacillus rhamnosus* GG	*Lactobacillus rhamnosus* GG administration during hospital stay was significantly associated with reduced cases of diarrhea.
Hempel et al. (2012) [22]	Participants of all ages (*n* = 11,811)	Systematic review and meta-analysis of randomized controlled trials	Antibiotic-associated diarrhea	*Lactobacillus*, *Bifidobacterium*, *Saccharomyces*, *Streptococcus*, *Enterococcus*, and/or *Bacillus* alone or in combination, using live microorganisms in probiotic or synbiotic preparations	The use of probiotics reduces antibiotic-associated diarrhea. There are research gaps concerning the specific probiotics with the greatest efficacy and the characteristics of patients receiving specific antibiotics.
Goldenberg et al. (2017) [23]	Participants of all ages (*n* = 8672)	Systematic review and meta-analysis of randomized controlled trials	*Clostridium difficile*-associated diarrhea	Various probiotics	Probiotics may be effective for the prevention of *Clostridium difficile*-associated diarrhea; however, the evidence is of moderate certainty.
Bai et al. (2022) [24]	Participants of all ages, with reference to children (*n* = NA)	Narrative Review	*Helicobacter pylori* infection	Various probiotics	Probiotics may excibit a beneficial impact on *H. pylori* eradication; however, more research is needed regarding the strains, dosages, duration. and safety of probiotic supplementation in clinical use.
Lü et al. (2016) [25]	Participants of all ages (*n* = 2306)	Meta-analysis of randomized controlled trials	*Helicobacter pylori* infection	Various probiotics	The treatment of *Helicobacter pylori* with probiotics may be effective regarding the improvement in eradication rates, the minimization of side effects incidence related to treatment, and the reduction in the most common clinical symptoms related to the condition.
McFarland et al. (2016) [26]	Participants of all ages (*n* = 2730)	Systematic review and meta-analysis	*Helicobacter pylori* infection	Multi-strain probiotics, mixture of *Lactobacillus acidophilus* and *Bifidobacterium animalis*	Multi-strain probiotics significantly improved eradication rates, prevented side effects and reduced antibiotic-associated diarrhea.
Shafaghi et al. (2016) [27]	Patients with a positive biopsy specimen for *H. pylori* (*n* = 76)	Randomized prospective double blind controlled trial	*Helicobacter pylori* infection	Synbiotics	The eradication rates were significantly higher in the group receiving synbiotics.
Pourmasoumi et al. (2019) [28]	*H. pylori*-infected patients aged 6–17 years old (*n* = 265)	Systematic review and meta-analysis	*Helicobacter pylori* infection	Synbiotics	*Helicobacter pylori* eradication rates were improved and side effects were reduced in the groups using synbiotics.
Ong et al. (2019) [29]	Newborn infants < 1 month old without diagnosis of infantile colic at recruitment (*n* = 1886)	Systematic review and meta-analysis	Infantile colic	Any probiotic, alone or in combination with a prebiotic	Probiotics did not prove more effective compared to placebo regarding infantile colic prevention, despite the reduced crying time in the probiotics group compared to placebo.
Ding et al. (2019) [30]	Children and adolescents 0 to 18 years (*n* = 829)	Rapid review of randomized controlled trials	Functional abdominal pain disorders	*Lactobacillus rhamnosus* GG, *Lactobacillus reuteri* DSM 17938, bifidobacteria and VSL3	The use of probiotics, particularly LGG, might reduce abdominal pain in children with IBS. The positive effects of the administration of other probiotics, including *L. reuteri* DSM 17938, i.e., pain reduction in patients with functional abdominal pain, IBS, and functional dyspepsia, are Inconsistent.
Ooi et al. (2019) [31]	Participants of all ages with reference to children (*n* = NA)	Narrative review of systematic reviews and meta-analyses	Irritable bowel syndrome (IBS)	Probiotics and prebiotics	The treatment of probiotics may improve IBS symptoms. A recommendation on specific species cannot be issued.
Fujimura et al. (2016) [32]	Infants aged 1–11 months old and 16S rRNA sequencing neonates, median age 35 days (*n* = 298)	Prospective study	Childhood atopy and asthma	Gut macrobiota bacteria	The high-risk group had lower relative numbers of certain bacteria, higher relative numbers of particular fungi, and a distinct fecal metabolome rich in pro-inflammatory metabolites. The dysbiosis of the neonatal gut microbiome results in CD4+ T–cell dysfunction, which is associated with childhood atopy.
Sestito et al. (2020) [33]	Children aged 0–2 years old (*n* = NA)	Narrative review	Allergy prevention in infants	Prebiotics and/or probiotics, and symbiotics	The overall preventive effect of prebiotics and probiotics regarding allergic diseases is unclear due to methodology heterogeneity in the studies examined. The impact of probiotics and/or prebiotics for the prevention of atopic dermatitis in infants at high risk is an exception.
Zhang et al. (2016) [34]	17 trials involving *n* = 2947 infants	Systematic review and meta-analysis of randomized controlled trials	Atopy and food hypersensitivity in early childhood	Probiotics	Probiotics administered prenatally and postnatally could reduce the risk of atopy and food hypersensitivity, especially when administered prenatally to pregnant mother and postnatally to children. No effect of probiotics administered prenatally or postnatally has been reported in atopy and food hypersensitivity
Cuello-Garcia et al. (2017) [35]	Infants and children aged 1–36 months old (*n* = 7832)	Systematic review and meta-analysis of randomized controlled trials	Allergy prevention	Prebiotic supplementation	Evidence on the effect of prebiotics on the risk of allergies is characterized by very low certainty.
Ng et al. (2019) [36]	Infants, children, and adolescents up to 16 years old with autism spectrum disorder (ASD) (*n* = 544)	Systematic review of clinical trials	Gastrointestinal (GI) complaints and behavioral issues	Prebiotics and probiotics	Limited efficacy has been reported regarding the use of prebiotics and probiotics in the control of GI or behavioral symptoms in children with ASD.
Ligezka et al. (2021) [37]	Infants, children, and adolescents aged 0–18 years old (*n* = 184)	Systematic review of randomized controlled trials, and cohort studies	Neuropsychiatric disorders	Probiotic	More studies are needed to determine whether gut dysbiosis leads to the development and/or contributes to the severity of mental disorders or whether gut dysbiosis is a result of other processes that accompany mental disorders.
McVey Neufeld et al. (2016) [38]	Adolescents (*n* = NA)	Review	Psychiatric illnesses	Gut macrobiota	Gut bacteria can influence the brain via the microbiota–gut–brain axis. Changes to the microbiota may contribute to the pathogenesis of psychiatric illness.
Ipar et al. (2015) [39]	Obese children and adolescents aged 4 to 17 years (*n* = 77). Control group: children and adolescents aged 5 to 17 years (*n* = 40)	Randomized controlled trial	Obesity	Synbiotic supplementation	Changes in weight, body mass index, and triceps skinfold thickness were higher in the group receiving the one-month synbiotic supplement than in the standard method group.
Dror et al. (2017) [40]	*n* = 13 adults, 17 children, and 23 infants participants iRCTs	Meta-analysis of randomized controlled trial (RCT)	Weight change	Probiotics, prebiotics, synbiotics, and antibiotics	Effects were opposite between adults and children, showing weight loss among adults and minor weight gains among children and infants taking mainly Lactobacillus probiotic supplements. There may be a role for probiotics in promoting weight loss in adults and weight gain in children; however, additional studies are needed.
Gomes et al. (2020) [41]	4 studies evaluated the gut microbiota of infants (*n* = 79), children, and adolescents (*n* = 201) with constipation. 8 placebo controlled randomized trials of probiotics/comparison in constipation in infants (*n* = 170)	Systematic review	Constipation	Probiotics	Despite the probiotics’ positive effects on certain characteristics of the intestinal habitat, there is still no evidence to recommend it in the treatment of constipation in pediatrics.
Scott et al. (2019) [42]	Children and adolescents up to 18 years (*n* = 3488)	Randomized controlled trial (RCT)	Acute otitis media (AOM)	Probiotics	Probiotics may prevent AOM in children not prone to AOM, but the inconsistency of the subgroup analyses suggests caution in interpreting these results. Probiotics decreased the proportion of children taking antibiotics for any infection.
Benic et al. (2019) [43]	Patients wearing orthodontic braces, mean age 14.9 ± 3.2 years, range 10–30 years (*n* = 64)	Randomized, triple-blind, placebo-controlled trial	Halitosis	Oral probiotics	Oral probiotic *S. salivarius M18* reduced the level of halitosis in patients wearing orthodontic braces but did not decrease their plaque or gingival indices. It was found that the regular use of oral probiotics did not influence plaque index and gingival index scores but resulted in a prolonged reduction in volatile sulfur compound levels.
Piqué et al. (2019) [44]	Participants of all ages, with reference to children (*n* = NA)	Review	Protection against enteropathogens and maintenance of intestinal barrier integrity.	Heat-killed (including tyndallized) probiotic bacteria (lactic acid bacteria)	Heat-killed bacteria or their fractions or purified components have key probiotic effects, with advantages versus live probiotics (mainly their safety profile), positioning them as interesting.
Morniroli et al. (2021) [45]	Term/preterm, healthy/ill infants and children (*n* = NA)	Review	Necrotizing enterocolitis and late-onset sepsis in preterm newborns, common childhood conditions (infections, allergies)	Postbiotics	Postbiotics have a positive effect on the development of the microbiota, intestinal maturity, and multiple Immunomodulating actions, which makes them particularly interesting in children.
Malagón-Rojas et al. (2020) [46]	Children younger than 5 years (*n* = 1740)	Systematic review of 7 RCTs	Common infectious diseases	Postbiotics: heat-killed *Lactobacillus acidophilus LB* and heat-inactivated *L. paracasei CBA L74*	Limited evidence to recommend the use of specific postbiotics for treating pediatric diarrhea and preventing common infectious diseases among children.
Mosca et al. (2019) [47]	Preterm infants (*n* = NA)	Review	Necrotizing enterocolitis	Postbiotics	Postbiotics may be a promising effective preventive strategy against necrotizing enterocolitis.
Lemoine et al. (2023) [48]	Infants (n = NA)	Review	Immune system	Pre-, pro-, syn-, and postbiotics in infant formulae	Positive health effects in infants in preventing infancy infections, in improving atopic dermatitis, or in accelerating food allergy remission.
Zagato et al. (2014) [49]	C57/BL6 mice (*n* = NA)	In vitro and in vivo study	Anti-inflammatory activity on dendritic cells and effects against colitis and an enteric pathogen	*Lactobacillus paracasei* CBA L74 metabolic products and fermented milk for infant formula	*L. paracasei CBA L74* fermented formula can provide immune benefits to formula-fed infants.
Athalye-Jape et al. (2017) [50]	Preterm infants (*n* = 2978)	Systematic review of randomized controlled trials (RCTs) and non-RCTs	Usual health conditions in preterm infants	*Bifidobacterium breve M-16V* was used as a probiotic	Significant benefits for late-onset sepsis, mortality, and postnatal age at full feeds (days). There were no adverse effects from *B breve M-16V.*

NA: not applicable.

## Data Availability

Not applicable.
